# Development of a latex microsphere-based lateral flow immunoassay for the diagnosis of schistosomiasis japonica

**DOI:** 10.1371/journal.pntd.0012742

**Published:** 2024-12-16

**Authors:** Emmanuel John Tabilin, Catherine A. Gordon, Yi Mu, Mario Jiz, Marianette Inobaya, Eleonor Avenido-Cervantes, Darren Gray, Mary Lorraine Mationg, Donald P. McManus, Thomas G. Egwang, Moses Adriko, Yasuhito Sako, Marcello Otake Sato, Megumi Sato, Hong You, Matthew Kelly, Pengfei Cai

**Affiliations:** 1 Molecular Parasitology Laboratory, QIMR Berghofer Medical Research Institute, Brisbane, Queensland, Australia; 2 Faculty of Medicine, The University of Queensland, Brisbane, Queensland, Australia; 3 Research Institute for Tropical Medicine, Department of Health, Manila, Philippines; 4 Population Health Program, QIMR Berghofer Medical Research Institute, Brisbane, Queensland, Australia; 5 Department of Applied Epidemiology, National Centre for Epidemiology and Population Health, Australian National University, Canberra, Australian Capital Territory, Australia; 6 Department of Immunology and Parasitology, Med Biotech Laboratories, Kampala, Uganda; 7 Vector Borne & Neglected Tropical Disease Control Division, Ministry of Health, Kampala, Uganda; 8 Asahikawa Medical University, Asahikawa, Japan; 9 Faculty of Medical Technology, Division of Global Environment Parasitology, Niigata University of Pharmacy and Medical and Life Sciences, Niigata, Japan; 10 Graduate School of Health Sciences, Niigata University, Niigata, Japan; 11 School of Biomedical Sciences, The University of Queensland, Brisbane, Queensland, Australia; George Washington University School of Medicine and Health Sciences, UNITED STATES OF AMERICA

## Abstract

**Background:**

Zoonotic schistosomiasis, caused by *Schistosoma japonicum*, is prevalent in China, the Philippines and Indonesia. Rapid point-of-care (POC) diagnostics are attractive and promising tools for evaluating the efficacy of intervention strategies for schistosomiasis control.

**Methodology:**

The diagnostic potential of five recombinant antigens was tested by enzyme-linked immunosorbent assay (ELISA) using sera from individuals with positive Kato-Katz (KK) results for *S*. *japonicum* (n = 28) and non-endemic controls (n = 12). A latex microsphere (LM)-based lateral flow immunoassay (LFIA) incorporating the recombinant SjSAP4 (rSjSAP4) was developed for the diagnosis of schistosomiasis japonica. The test conditions including diluent, dilution factor and reaction time, were optimised for the developed LFIA. Under the optimised conditions, serum samples from individuals living in a barangay endemic for *S*. *japonicum* (n = 549) and non-endemic controls (n = 50) were tested with the established LFIA cassettes. The results were imaged by a smartphone and analysed by the ImageJ program. The intensity ratio of the test line to the control line (T/C ratio) was calculated for each cassette.

**Main findings:**

ELISA confirmed that rSjSAP4 was the optimal candidate for serological diagnosis of schistosomiasis japonica. Under optimal testing conditions, the developed LFIA strips had a sensitivity of 80.6% and a specificity of 98.0% at a cut-off T/C ratio of 0.1031. Moreover, the results of the LM-based LFIA was positively correlated with those obtained from the rSjSAP4-ELISA (r = 0.8270, 95% CI, 0.7990–0.8514; *p* < 0.0001). The schistosomiasis prevalence determined by the LFIA strips was about 1.8 times greater than that obtained with the 6-slide KK procedure performed on three stool samples.

**Conclusions/Significance:**

The developed LFIA represents a POC diagnostic tool that is suitable for onsite screening of human *S*. *japonicum* infection with minimal equipment needed. The established immunochromatographic assay complies with most of the WHO’s ASSURED criteria for POC diagnostics.

## Author summary

Schistosomiasis remains a significant global public health concern, with over 230 million people affected. Integrated multicomponent interventions targeting the various aspects of schistosomiasis epidemiology represent promising efforts that may eventually lead to the disease elimination. Rapid and accurate point-of-care diagnostics (POCs) are crucial tools for informing the effective implementation of these elimination efforts. In this study, we developed a latex microsphere (LM)-based lateral flow immunoassay (LFIA) by incorporating rSjSAP4 for the rapid diagnosis of schistosomiasis japonica. Serum samples collected from subjects living in an area endemic for *S*. *japonicum* and non-endemic controls were tested with the established LFIA strips. In the individuals who tested positive by the Kato-Katz method, the LM-based LFIA cassette showed 80.6% sensitivity and 98.0% specificity. When the entire Philippines cohort was tested, the developed LFIA showed a high consistency with the rSjSAP4-ELISA. The schistosomiasis incidence deduced from the developed LFIA and rSjSAP4-ELISA was about 1.8 times higher than that determined with the KK procedure. The findings obtained here further indicate that the rSjSAP4-incorprated LFIA meets most of the ASSURED criteria set by the WHO for POC diagnostics, representing an effective diagnostic tool for the screening of *S*. *japonicum* infection in rural endemic areas with limited resources.

## Introduction

Schistosomiasis is a severely debilitating and potentially fatal tropical disease. Caused by helminth parasites of the genus *Schistosoma*, it affects more than 230 million people worldwide [1]. *Schistosoma mansoni* (Africa and South America), *S*. *haematobium* (Africa), and *S*. *japonicum* (Asia) are the three major schistosome species infecting humans. Among them, *S*. *japonicum* is a zoonotic parasite that can use more than 40 mammalian as reservoir hosts [2,3]. The control of schistosomiasis focuses on reducing disease through periodic, large-scale population preventive treatment with praziquantel, referred to as mass drug administration (MDA) [4]. Both the prevalence and infection intensity of the disease have declined in many endemic areas primarily due to the implementation of MDA as part of national control programs, although other more integrated methods have been used [5]. In China, for example, bovines, a major reservoir host for transmission of *S*. *japonicum*, were removed from endemic areas and replaced with tractors [6]; however, this method is not sustainable in the Philippines. The sustainability of schistosomiasis control programs is limited by multiple factors, such as repeated infection—MDA does not prevent re-infection [1], climate change, which may extend or change endemic zones [7,8], and the zoonotic nature of schistosomiasis in Asia.

In the low endemicity areas (LEAs) of schistosomiasis, there is a need to develop and implement new, cost-effective and accurate diagnostic tools for rapid mapping and monitoring of schistosomiasis [9]. While conventional parasitological diagnostic methods (e.g., the microscopy-based Kato-Katz (KK) procedure and urine filtration) are labor- and time-intensive, and have limited sensitivity in the detection of schistosomiasis in LEAs, point-of-care (POC) diagnostics are becoming ideal tools for the disease control and elimination. To aid in the development of POCs, the WHO has compiled the ASSURED criteria, which have now been updated as REASSURED [10].

POC diagnostics of schistosomiasis can be divided into three main categories: immunological, molecular-based, and mobile phone microscopes [11]. Molecular-based POCs aim to take advantage of rapid DNA extraction (e.g., DNA dipstick) [12] and isothermal nucleic acid amplification methods [e.g. loop-mediated isothermal amplification (LAMP) and recombinase polymerase amplification (RPA)], and/or CRISPR-based techniques to amplify signals [13,14]. While promising results have been reported, these are mostly still in the development phase, with cost being a major concern. Mobile phone microscopes are portable and battery-powered devices designed to analyse samples at the point of collection [15]; such devices for schistosomiasis have been suggested to be ideal tools for rapid screening of *S*. *haematobium infection* in community- or school-based settings [16,17]. The immunological POCTs could be further subcategorised into antigen detection (AgD) and antibody detection (AbD)-based tests [11]. AgD-based POC tests for schistosomiasis use specific monoclonal antibodies (mAbs) to capture *Schistosoma* gut-associated proteoglycan components known as circulating anodic antigens (CAAs) or circulating cathodic antigens (CCAs) [18]. CAA-targeting POCTs such as POC-CCA and POC-ECO have been used to diagnose *S*. *mansoni* infection by testing urine samples [19,20]. However, these assays suffer some pitfalls, such as cross-reactivity and specificity problems, as well as the issue in reading ‘trace’ results [21,22]. In addition, the POC-CCA has shown limited applicability for the diagnosis of other *Schistosoma* species [22–24]. The up-converting phosphor-lateral flow CAA (UCP-LF CAA) assay can detect all *Schistosoma* species quantitatively, exhibiting more accurate diagnostic performance than the POC-CCA [25]. The UCP-LF CAA is currently not available as a commercial test [26], and the assay has equipment and reagent requirements that make it less than ideal as a POC [27]. The AbD-based POCTs developed for the diagnosis of schistosomiasis typically use crude worm or egg antigens as targets, causing potential cross-reactions with other helminths [11]. Nevertheless, three recombinant antigen-based lateral flow immunoassays (LFIAs) have been developed for the detection of schistosome infections [28,29].

By incorporating SjSAP4, we previously developed a colloidal gold immunochromatographic assay (GICA), which showed a sensitivity of 83.3% and absolute specificity in the detection of KK positive individuals recruited from Northern Samar, the Philippines [30]. For developing LFIA, colloidal gold nanoparticles (Au NPs) labelling is the easiest, fastest, and most conventional method to produce conjugates for visualisation and the results can be determined by the naked eye. However, various nanomaterials with characteristic optical properties, such as fluorescent/colored latex microspheres (LMs), carbon NPs, quantum dots (QDs), and up-converting phosphor/NPs [31–38], are also used as probes for LFIA development. LM-based LFIA cassettes have several advantages over AuNPs-based cassettes, such as increased sensitivity and stability [39–41]. In this study, we aimed to develop an LM-based LFIA for the diagnosis of schistosomiasis japonica by incorporating rSjSAP4 and assess its performance with serum samples collected from a rural area endemic for *S*. *japonicum* in Leyte Province, the Philippines.

## Materials and methods

### Ethics statement

Ethical approval for this study was obtained from the QIMR Berghofer Medical Research Institute (QIMRB) Animal (Project: P3706) and Human Ethics Committees (Projects: P3700 and P3797), the Institutional Review Board of the Research Institute for Tropical Medicine (RITM) (ID No. 2022–16), the Philippines, and the Institutional Review Board, Vector Control Division, Ministry of Health, Uganda (IRB No. VCDREC160). Informed written consent was obtained from each participant (for individuals aged 18 years or under, written consent was obtained from their legal guardians). The study was conducted in accordance with the Australian Code for the Care and Use of Animals for Scientific Purposes (8th edition, 2013) and the National Statement on Ethical Conduct in Human Research (July 2018).

### Animals and parasites

Experimental *S*. *japonicum* infection in a mouse model was performed as previously described [42]. Briefly, *S*. *japonicum* cercariae (Philippine strain, *SjP*) were shed from parasite-infected *Oncomelania hupensis* snails under light stimulus. Eight-week-old female BALB/c mice (n = 3) were infected percutaneously with 80 ± 5 *S*. *japonicum* cercariae. The mice were euthanised at 7 weeks post-infection (p.i.). Blood samples were collected via the tail vein and serum was separated by centrifugation at 4,000 rpm for 10 min. Serum samples were collected from naive mice using the same method and served as controls. Serum samples were collected from a New Zealand white rabbit infected with approximately 1,000 *S*. *japonicum* cercariae (Chinese strain, *SjC*) prior to infection and at 6 weeks p.i.

### Study cohort, sample collection, processing and storage

During Oct 2022, study participants (aged 2–65 years) living in the endemic village of Ekiran in Leyte Province, the Philippines, were consented and enrolled (n = 599). Individuals who had lived in the study area for less than two years were excluded. For each participant, three stool samples were sought on different days. For blood sample collection, 10 ml of blood (1 to 2 ml from children aged 2–5 years) was collected from each individual. Blood samples were allowed to clot for at least 30 min followed by centrifugation at 1,500 × *g* for 10 min. The separated serum was aspirated and aliquoted into 2 ml cryotubes, and stored at –80°C. Serum samples (n = 50) collected from a rural area non-endemic for schistosomiasis in Kabale District, Uganda, served as negative controls.

### Parasitological detection

Two KK slides were prepared for each stool sample. The stool sample was sieved by pressing it through a thin gauze. Approximately 50 mg of sieved stool was placed on a microscope slide using a volume template. A piece of cut green cellophane soaked in glycerol was placed over the sample and then pressed firmly to spread the faeces. The slides were left for at least 30 min and then placed in a slide box for storage. The KK slides were prepared by laboratory staff and read by trained microscopists at RITM, the Philippines. The KK results were expressed as the number of eggs per gram (EPG) of faeces.

### Recombinant proteins

Four recombinant proteins, rSj23-LHD (GenBank Accession No. XBP28865.1), rSjSP13-V1 (GenBank Accession No. UTN00450.1), rSjSAP4 (GenBank Accession No. UTN00449.1), and rSjSAP5 (GenBank Accession No. XBP28864.1) were produced in a previous study [43]. To produce the recombinant protein SjSP13-V2 (GenBank Accession No. UTN00451.1), primers (forward: 5’-AAGGATCCCTTGAAAATTCTGTGTCACC-3’ and reverse: 5’-CTGCTCGAGAATAGTGAATTGAACTAGAAACTTC-3’) were used to amplify a specific region of the target from cDNA isolated from *SjP* adult worms by PCR. The DNA fragment was subsequently cloned into the pET-28a plasmid via the BamHI and XhoI digestion sites. After transformation into the competent *Escherichia coli* strain JM109, the recombinant plasmids were extracted from multiple colonies and sequenced by the Sanger method. One correct plasmid was transformed into the competent *E*. *coli* strain BL21 (DE3). The expression of rSjSP13-V2 was induced by 0.5 mM isopropyl β-D-1-thiogalactopyranoside (IPTG) at 37°C for 4 h. The rSjSP13-V2 protein was purified under denaturing conditions as previously described [43].

### Enzyme-linked immunosorbent assay (ELISA)

Indirect ELISA tests were performed as previously described [43,44]. Wells of MaxiSorp 96-well plates (Thermo Fisher Scientific, Australia) were coated with 100 μl of 1 μg/ml recombinant protein in coating buffer and incubated overnight at 4°C. The plate contents were discarded and the plates were patted dry on tissue paper, then blocking buffer (130 μl) was added to each well and allowed to block for 1 h at 37°C. Serum samples were diluted (1:100) with 1% BSA blocking buffer (PBS with 0.05% Tween 20, 1% BSA). The diluted sera (100 μl) were then added to their corresponding wells in duplicate. The plates were then incubated at 37°C for 1 h. The secondary antibody, a mouse monoclonal anti-human IgG Fc specific)-biotin antibody (Sigma-Aldrich Co, MO, USA) was diluted with the blocking buffer and added to each well (1:20,000, 100 μl/well). After incubation at 37°C for 1 h, the streptavidin-HRP (BD Biosciences, CA, USA) was diluted with the blocking buffer and added to each well (1:10,000,100 μl/well). The plates were then incubated at 37°C for 30 min prior to adding 100 μl of TMB substrate mixture for color development, which was stopped by adding 50 μl of 2 M H_2_SO_4_ to the wells. For each plate, the optical density at a wavelength of 450 nm (OD_450nm_) was measured using a microplate reader (PowerWave XS2, BioTek, VT, US). After incubation with human sera, the plates were washed with PBST 5 times between each step. ELISA cut-off value was calculated as 2.1× the mean OD_450nm_ of all the negative controls with outliers removed.

### Preparation of LFIA cassettes

**Fig 1:**
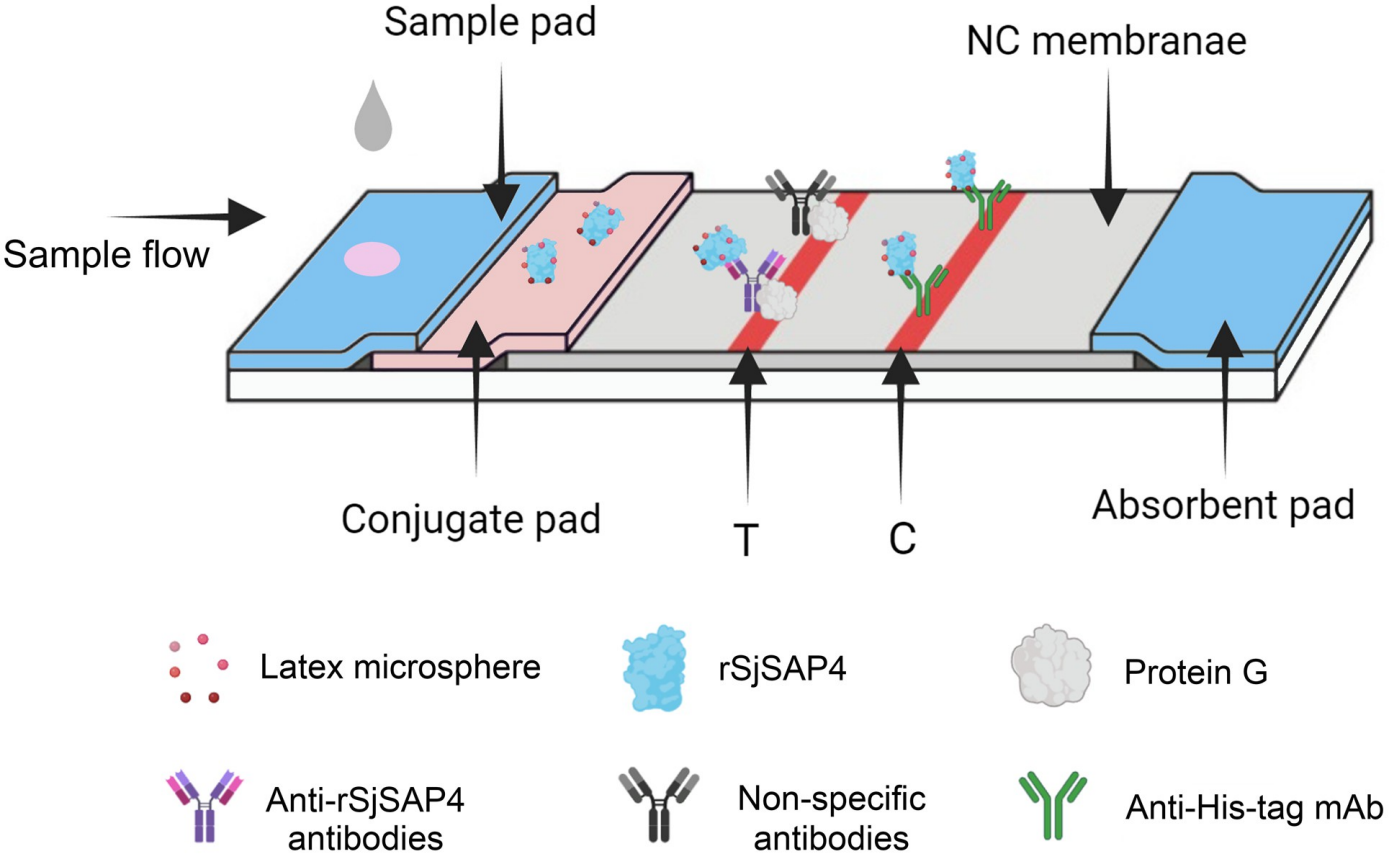
Schematic illustration of the LM-based LFIA cassette for the detection of *S*. *japonicum* infection. The cassette implements a design that incorporates the LM-labelled rSjSAP4 in the conjugate pad, instead of on the test (T) line as in a typical AbD-based LFIA. As a positive serum sample flows into the conjugate pad, specific anti-rSjSAP4 Abs present in the sample bind the LM-conjugated rSjSAP4 to form immunocomplexes, which can be further captured by the protein G immobilised on the T line. On the control (C) line, unbound LM-labelled rSjSAP4 protein is captured by a monoclonal Ab specific to the histidine tag. A positive reaction is determined by the development of both reddish T and C lines. If the C line does not appear, the test is considered invalid and must be repeated.

An LM-based LFIA cassette was designed (Fig 1) and then fabricated by Zoonbio Biotechnology (Nanjing, China) as previously described [28], with modifications to the conjugate labelling method. In the current cassette, red LMs (300 nm) were used in place of colloidal AuNPs to conjugate rSjSAP4.

### LFIA test and analysis

To determine the optimal serum diluent, a pooled serum sample mixed from *S*. *japonicum*-infected individuals (n = 3) was diluted 1:10 with five buffers, including PBS, PBS with 0.05% Tween 20, PBS with 1% BSA, 0.9% NaCl, and 2.5% sucrose with 1% Tween-20. To test the LFIA cassette, 50 μl of each diluted serum sample was dispensed into the sample well of the cassette. The strip results were captured by a smartphone after 10 min and every 5 min thereafter, until 25 min.

To determine the best serum dilution ratio, PBS and 2.5% sucrose with 1% Tween-20 were selected as diluents. Serum samples from KK-positive [KK (+)] individuals (n = 3) with different levels of anti-rSjSAP4 IgG antibodies and non-endemic control individuals (n = 3) were diluted at different ratios (1:10, 1:100, 1:1000 and 1:10000). Each diluted serum sample (50 μl) was then loaded on the LFIA cassette. The results were captured by a smartphone at 20 min after sample loading.

For the formal test of the LFIA cassettes, PBS was used as the diluent and 1:10 was used as the dilution ratio. The LFIA results were captured by a smartphone (iPhone 8) at 25 min after sample loading. For result analysis, all the images were uploaded to a computer and profile intensity plots of the test images were generated using the Java-based image-processing program ImageJ 1.53 to quantify intensity of developed T and C lines. Quantitative results were produced by introducing a T/C ratio, which was defined as the intensity ratio between the T line and the C line, for each test.

### Statistical analysis

Statistical analyses were performed using GraphPad Prism version 10.2.3 software (GraphPad Software, Inc., San Diego, CA, USA), with the significant *p*-value set as <0.05. For ELISA and LFIA tests, a comparison between the KK (+) and control samples was performed using the Mann–Whitney *U* test. The Kruskal–Wallis test followed by Dunn’s comparison was used to determine the difference in T/C ratio between different groups. Receiver operating characteristic (ROC) curve analysis was performed to measure the overall performance of the developed diagnostics. The cut-off T/C ratio for the LFIA was set as the maximum Youden index. Correlation between the rSjSAP4-ELISA and LFIA results was assessed using the Spearman’s rho. The qualitative agreement between the rSjSAP4-ELISA and LFIA tests was measured by the kappa statistic (https://www.graphpad.com/quickcalcs/kappa1/).

## Results

### Diagnostic performance of five *S*. *japonicum* recombinant antigens determined by indirect ELISA

**Fig 2:**
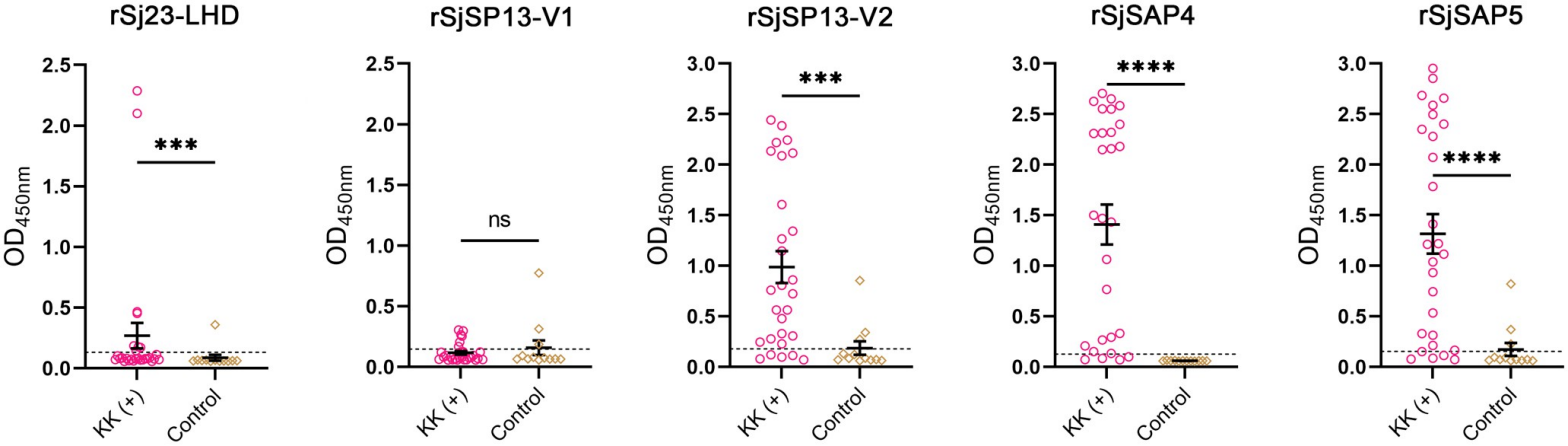
IgG antibody levels against five *S*. *japonicum* recombinant antigens in sera from KK (+) individuals and non-endemic controls. Statistical significance between the KK (+) (n = 28) and control (n = 12) samples was determined using the Mann–Whitney *U* test (ns–no significant difference; ***, *p* < 0.001; ****, *p* < 0.0001). Dashed lines correspond to OD_450nm_ cut-off value calculated as 2.1× the mean OD_450nm_ of control samples with outlier datasets removed.

Indirect ELISA tests were performed to detect the IgG responses to five *S*. *japonicum* recombinant antigens, including the tegument-associated protein Sj23-LHD and four saposin proteins SjSP13-V1, SjSP13-V2, SjSAP4, and SjSAP5 (Fig 2). Mean IgG levels against all except SjSP13-V1 were significantly higher in the KK (+) individuals (n = 28) compared with those from the controls (n = 12). The highest seropositivity among KK (+) individuals was achieved with the rSjSAP4-ELISA with a sensitivity of 85.7%, and no positivity was detected in non-endemic sera. Both the rSjSP13-V1-ELISA and the rSjSAP5-ELISA yielded a high sensitivity of 82.1%, but three control samples had positive reactions resulting in a specificity of 75% for these assays. In contrast, the rSj23-LHD-ELISA and rSjSP13-V1-ELISA had poor diagnostic performance, detecting only 28.6% and 21.4% of infected samples, respectively.

### Optimisation of testing parameters for the LM-based LFIA

**Fig 3:**
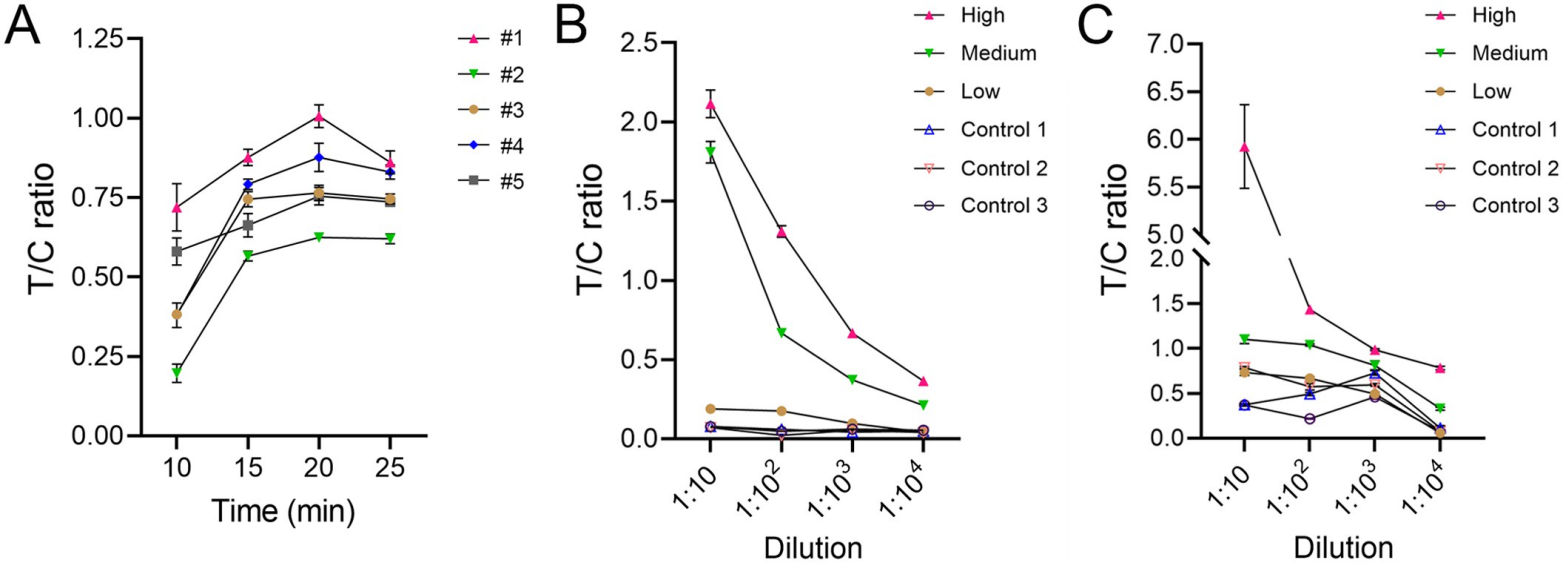
Determination of optimal testing parameters for the developed LFIA. (A) Three serum samples from *S*. *japonicum*-infected individuals with different anti-rSjSAP4 IgG levels as per the OD values from the rSjSAP4-ELISA, were equally pooled. The pooled serum was diluted with five different diluents (1:10) and tested with the LFIA cassettes. #1, PBS; #2, PBS with 0.05% Tween-20 (PBST); 3#, PBS with 1% BSA; 4#, 0.9% NaCl; #5, 2.5% sucrose with 1% Tween-20. The results captured at 10, 15, 20 and 25 min after sample loading are presented. The LFIA cassettes were tested with (B) PBS- or (C) the sucrose diluent-diluted serum samples from KK (+) individuals (n = 3) with different anti-rSjSAP4 IgG levels and non-endemic controls (n = 3) over different dilution factors. Panels A-C, data are presented as mean ± SEM from two independent analyses. Panels B and C, the results obtained at 20 min after sample loading are presented. High, medium, and low anti-rSjSAP4 IgG levels were determined by the rSjSAP4-ELISA.

Three serum samples from KK (+) individuals with different anti-rSjSAP4 IgG levels according to the rSjSAP4-ELISA were equally pooled. The pooled serum was then diluted 1:10 with five different buffers, including PBS, PBS with 0.05% Tween-20 (PBST), PBS with 1% BSA, 0.9% NaCl, and 2.5% sucrose with 1% Tween-20. Each diluted serum sample was tested with the LFIA cassette, and the results were imaged at different time points and analyzed with the ImageJ program to generate T/C ratio curves over time (Fig 3A). For all the diluents tested, the T/C ratio peaked at 20 min after sample loading, with the sample diluted with PBS exhibiting the highest T/C ratio over different time points (Fig 3A). In terms of the intensity of the T line, the pooled serum sample diluted in the sucrose diluent yielded the highest value.

Two diluents, PBS and 2.5% sucrose with 1% Tween-20, were further tested for the optimal dilution factor using sera from three infected individuals showing low, medium and high anti-rSjSAP4 IgG levels, respectively, and three non-endemic controls. When PBS was used as the diluent, the T/C ratios of infected samples decreased with increasing dilution factors whereas those of the non-endemic control samples remained consistently low (Fig 3B). Sera with high and medium anti-rSjSAP4 IgG levels were positive up to the highest dilution tested (1:10,000); while the serum with low anti-rSjSAP4 IgG level was positive up to a dilution of 1:100 (Fig 3B, a test was determined to be positive when the T/C ratio was greater than 2.1 times the mean T/C ratio of the control samples tested at the same dilution). When the samples were diluted with 2.5% sucrose containing 1% Tween-20, the T/C ratios decreased for the three KK (+) serum samples with increasing dilution factors (Fig 3C). Notably, the control serum samples diluted in the sucrose diluent also presented relatively high T/C ratios at dilutions of 1:10, 1:100 and 1:1000 (Fig 3C), indicating a non-specificity issue caused by the diluent. These results indicate that 1:10 is an optimal dilution factor, and PBS is the optimal diluent for testing the developed LFIA strips.

### Formal test of the developed LFIA cassette

**Fig 4:**
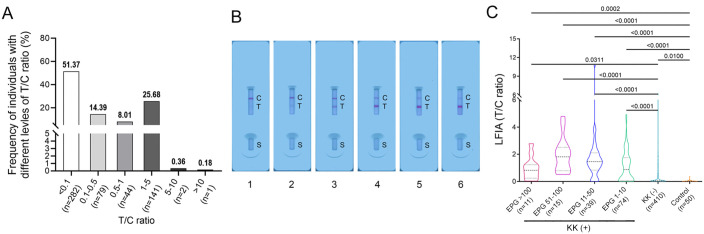
T/C ratio analysis for the developed LFIA cassettes. (A) Frequency of individuals (those from a barangay endemic for *S*. *japonicum*, n = 549) with different levels of T/C ratio. (B) Representative LFIA cassettes showing different levels of T/C ratio. Lanes 1–6, cassettes displaying a T/C ratio that falls in the ranges of 0–0.1, 0.1–0.5, 0.5–1, 1–5, 5–10, and >10, respectively. C, control line; T, test line; S, sample well. (C) Truncated violin plots showing the distribution of T/C ratio in the different KK (+) subgroups (EPG >100, n = 11; EPG 51–100, n = 15; EPG 11–50, n = 39; EPG 1–10, n = 74), KK (-) individuals (n = 410) and non-endemic controls (n = 50). The horizontal black dashed lines indicate median values, and the horizontal black dotted lines represent the interquartile range (IQR) of the data. *p* values were calculated using the Kruskal–Wallis test followed by Dunn’s comparison. The results obtained at 25 min after sample loading are presented. EPG, eggs per gram of faeces.

The LFIA cassettes were further tested with sera from KK (+) (n = 139), KK-negative [KK (-)] individuals (n = 410) and non-endemic controls (n = 50) under optimised testing conditions (1:10 dilution with PBS). In the cohort living in an endemic area of *S*. *japonicum* infection, most individuals had a T/C ratio <0.1 (51.37%), followed by those had a T/C ratio within the range of 1–5 (25.68%) (Fig 4A). In addition, 14.39% and 8.01% of the participants showed a T/C ratio falling within the ranges of 0.1–0.5 and 0.5–1, respectively; whereas only 0.36% and 0.18% of the individuals had a T/C ratio between 5–10 and greater than 10, respectively (Fig 4A). Fig 4B shows representative LFIA cassettes displaying a T/C ratio that falls in the ranges of 0–0.1, 0.1–0.5, 0.5–1, 1–5, 5–10, and >10, respectively. The differences in T/C ratio between the controls and the target cohort stratified by infection status and intensity were assessed. The T/C ratios were significantly greater in the subgroups with EPG > 100 (n = 11, *p* = 0.0002), EPG 51–100 (n = 15, *p* < 0.0001), EPG 11–50 (n = 39, *p* < 0.0001), EPG 1–10 (n = 74, *p* < 0.0001), and the KK (-) individuals (n = 410, *p* = 0.01) than those in the non-endemic controls (n = 50) (Fig 4C). In the target cohort, all KK (+) subgroups presented greater median T/C ratios than that from the KK (-) group (*p* = 0.0311, *p* < 0.0001, *p* < 0.0001 and *p* < 0.0001 for subgroups with EPGs >100, 51–100, 11–50 and 1–10, respectively) (Fig 4C). There were no differences in T/C value among different KK (+) subgroups.

### Diagnostic performance of the developed LFIA cassette

**Fig 5:**
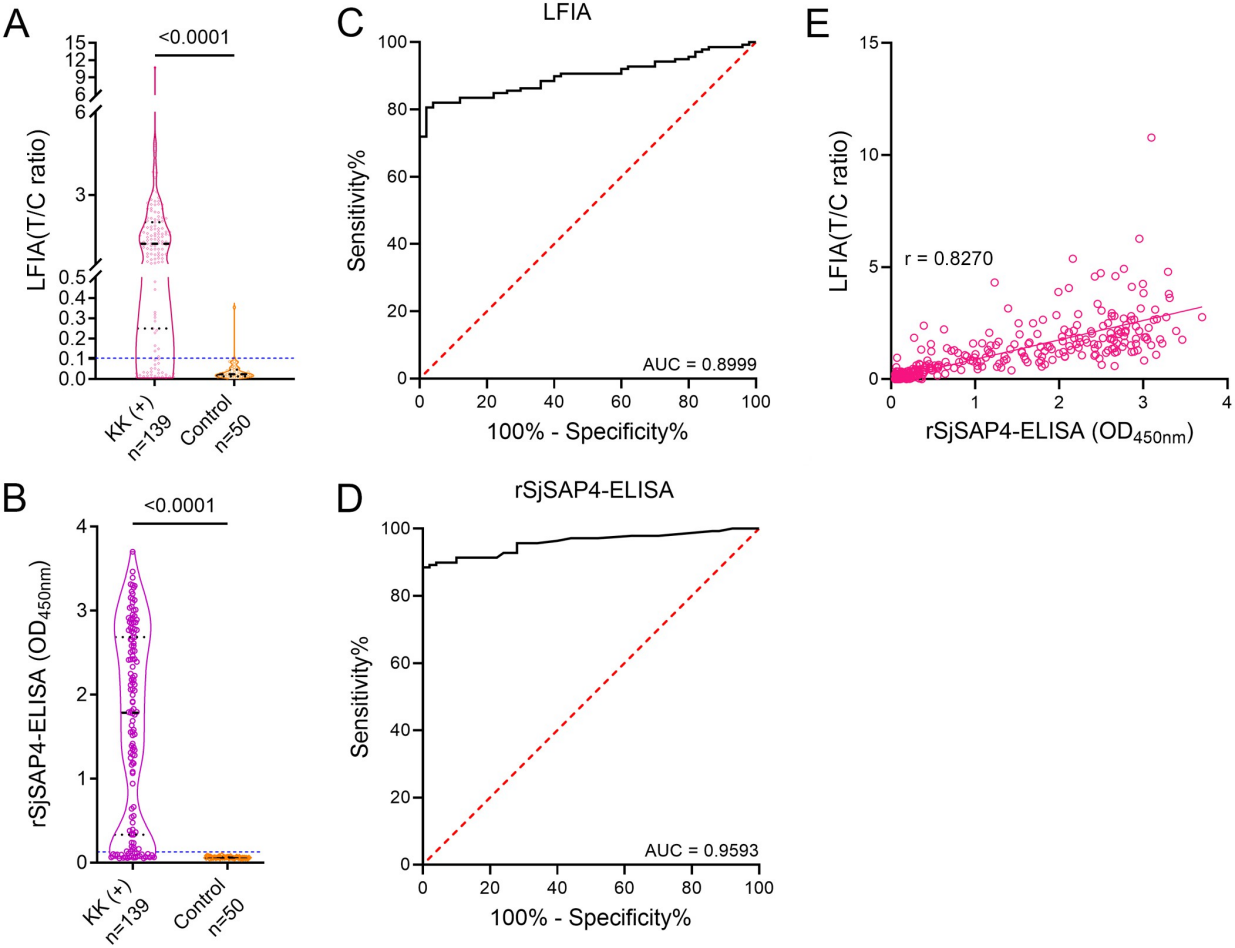
Diagnostic performance of the developed LFIA cassette. Truncated violin plots showing (A) the T/C ratios from the LFIA test and (B) the OD values from the rSjSAP4-ELISA performed on the serum samples from the KK (+) individuals (n = 139) and non-endemic controls (n = 50). The blue dashed line corresponds to the cut-off values. Statistical significance was determined using the Mann–Whitney *U* test. ROC curve analysis showing the performance of (C) the LFIA cassette and (D) rSjSAP4-ELISA in discriminating KK (+) individuals from controls. (E) Correlation between the LFIA and rSjSAP4-ELISA was assessed using Spearman’s correlation coefficient (*p* < 0.0001). The results captured at 25 min after sample loading are presented for the developed LFIA.

By testing samples from the KK (+) individuals (n = 139) and non-endemic controls (n = 50), the LFIA exhibited a sensitivity of 80.6% and a specificity of 98.0% at a cut-off T/C ratio of 0.1031 (Fig 5A). The rSjSAP4-ELISA tested with the same serum samples showed 82.0% sensitivity and 100.0% specificity (Fig 5B). ROC curve analysis for the LFIA and rSjSAP4-ELISA revealed area under the ROC curve (AUC) values of 0.8999 (95% CI, 0.8569–0.9428, *p* < 0.0001) and 0.9593 (95% CI, 0.9334–0.9851, *p* < 0.0001), respectively (Fig 5C and 5D). In the whole cohort, 266 and 248 serum samples were positive as per the developed LFIA and rSjSAP4-ELISA tests, respectively (Table 1). The agreement between the LFIA and rSjSAP4-ELISA results was substantial, with a kappa value of 0.795 (95% CI, 0.745–0.846) (Table 1). Similarly, the T/C ratios from the LFIA tests and the OD values from the rSjSAP4-ELISA were significantly positively correlated (*p* < 0.0001), with an r value of 0.8270 (95% CI, 0.7990–0.8514) (Fig 5E).

**Table 1:** Agreement analysis for the LFIA and rSjSAP4-ELISA tests performed on serum samples (n = 549) collected from a barangay endemic for *S*. *japonicum*.

		rSjSAP4-ELISA			Kappa index (95% CI)
		Positive	Negative	Total	
LFIA	Positive	229	37	266	0.795 (0.745–0.846)
	Negative	19	264	283	
	Total	248	301	549	

CI: Confidence interval.

### Applicability of the LFIA cassette across *S*. *japonicum* strains and cross-reactivity test

**Fig 6:**
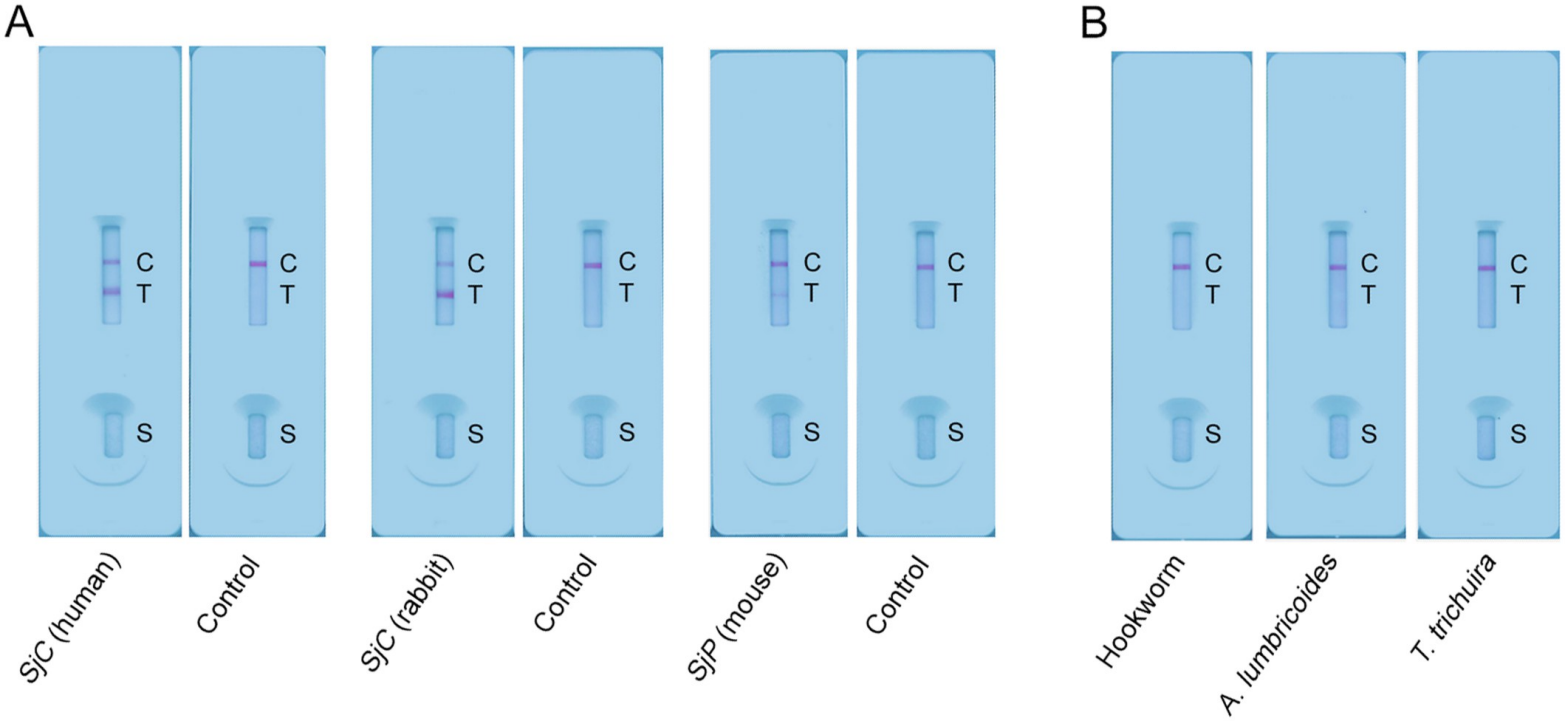
Applicability of the developed LFIA strip in the detection of different *S*. *japonicum* strains and cross-reactivity analysis of the immunochromatographic assay. (A) LFIA cassettes were used to detect *S*. *japonicum* infection across *SjC* and *SjP* strains. (B) Potential cross-reactivity with other helminth infections was assessed for the established LFIA cassette using the serum samples from individuals infected with hookworm, *A*. *lumbricoides* or *T*. *trichuira*. C, control line; T, test line; S, sample well.

The developed immunochromatographic cassette was performed on a serum sample from a human subject infected with the *SjC* (Fig 6A), demonstrating its applicability in the detection of *SjC* infection. Further, the cassettes were evaluated with a serum sample from a *SjC-*infected rabbit (6 weeks pi) and a pooled serum sample from *SjP-*infected mice (n = 3, and 7 weeks pi), which presented T/C values of 3.439 and 0.2384, respectively (Fig 6A), indicating the compatibility of the LFIA in detecting *S*. *japonicum* infection in non-human mammalian hosts. In addition, the developed LFIA strips were tested using sera from Uganda individuals infected with hookworm, *A*. *lumbricoides* and *T*. *trichuira*, and showed no cross-reactivity with these helminth species (Fig 6B).

## Discussion

*S*. *japonicum* remains highly endemic in a number of areas in the Philippines despite decades of control efforts centered on case management and preventative chemotherapy with praziquantel through MDA [6,45]. Integrated controls aimed at eliminating the disease in humans, animals and the environment have been initiated in the Philippines by the Department of Health (DOH) through the Schistosomiasis Control and Elimination Program (SCEP), addressing to the complex epidemiology of the disease [46]. One of these crucial efforts is to develop affordable, accurate and field-applicable diagnostics for screening, monitoring and surveillance [46]. Although field-friendly, the traditional KK procedure has been proven to be insufficiently sensitive to identify the true infection status in individuals with low schistosome infection burden [9,47,48]. The development of cost-effective and accurate POC diagnostics, which has been given priority in the SCEP Strategic Plan [49], has the potential to meet this need.

The current study aimed to develop an AbD-based LFIA for the diagnosis of *S*. *japonicum* infection by targeting recombinant antigens, where the identification of sensitive and specific antigens is a key step. We evaluated the diagnostic applicability of five *S*. *japonicum* recombinant antigens by ELISA tests. The sensitivity of the rSjSAP4-ELISA in the present study (85.7%) (Fig 2) is in agreement with that reported in our previous study (84.3%) involving a cohort in Northern Samar, the Philippines [43]. Meanwhile, the rSjSAP4-ELISA retained high specificity (100%) in the present study as previously observed [43]. The rSjSAP5-ELISA and rSjSP13-V2-ELISA were also highly sensitive, identifying more than 80% of the *S*. *japonicum*-infected individuals, but showed positivity in some control samples, resulting in a specificity of 75% for both assays. In addition, the rSj23-LHD-ELISA and rSjSP13-V1-ELISA showed poor performance in sensitivity. These results further confirmed that rSjSAP4 is the most suitable antigen for the detection of *S*. *japonicum* infection, with considerable sensitivity and specificity.

We have previously reported the development and assessment of an rSjSAP4-incorporated GICA using AuNPs as the detector probe [28,30]. Typically, as probes to be conjugated in LFIAs, the LMs are more sensitive, specific, stable and eco-friendly than AuNPs [40,48,50]. Here, we developed an LFIA with red LMs instead of AuNPs as the colorimetric detector using a similar format as adopted in the previously developed GICA. The optimisation of test conditions for the developed LFIA validated PBS as the optimal diluent based on the results read at different time points, with the optimal read time being 20 min as observed peak T/C ratios in testing a pooled serum sample (Fig 3A). With an optimal cut-off value, the LFIA had a sensitivity of 80.6% and specificity of 98.0%, exhibiting a similar diagnostic performance with the previously developed GICA strip, which has a sensitivity of 83.3% and absolute specificity [30]. As expected, the LFIA cassette showed applicability in testing individuals infected with *SjC* as SjSAP4 orthologues between *SjP* and *SjC* share 99% identity.

A number of methods/tools have been used to interpret strip results, such as the naked eye examination, use of interpretation reference cards [51], strip readers [52,53], and image processing programs, such as Image Studio Lite [54] and ImageJ [28]. Direct interpretation of results by the naked eye is simple and straightforward but can be subjective due to the differences in readers’ visual acuity and/or training, particularly in interpreting trace results [55]. The use of a color interpretation card as an auxiliary reference for the result readout converts the assay to a semi-quantitative test, yet a visual judgement is still engaged in the process. In contrast, quantification of color intensity by a chromogenic test reader or image processing algorithms can enable the assay to be accurately interpreted. In this study, the LFIA results were captured by a smartphone and further analysed by the ImageJ [23]. A T/C ratio was then introduced to eliminate inter-reader variability and convert the assay results into quantitative data, which can minimise the system errors such as differences in sample absorbance rate. This approach also enabled us to assess the correlation between the LFIA and rSjSAP4-ELISA results, which was significantly positive (r = 0.8270, *p* < 0.0001), indicating the applicability of the LFIA as a field-friendly alternative to ELISA without the need for sophisticated equipment.

Two major designs have been used in the development of AbD-based LFIA cassettes for the diagnosis of schistosomiasis. The first design, such as adopted by Shen *et al* [56] and Xu *et al* [57], can be applied for the detection of schistosomiasis in both humans and domestic animals, whereas the second format was primarily designed for the detection of human schistosomiasis such as developed in the previous studies [29,51]. Theoretically, the LFIA cassette format designed in this study also enables the detection of anti-rSjSAP4 IgG antibodies across mammalian hosts. Indeed, the developed LFIA showed applicability in the detection of *S*. *japonicum* infection in rabbit and mouse, however, varied T/C values were observed between the two animal models (Fig 5A), potentially due to the differences in infection intensity and/or binding affinity of protein G with Abs originating from different mammals [58]. More than 40 mammalian animals such as bovines, goats and pigs, serve as reservoir hosts for *S*. *japonicum* [1], playing a key role in the transmission of schistosomiasis japonica. The applicability and reliability of the developed LFIA for the detection of *S*. *japonicum* infection in other reservoir hosts, need to be validated before its field application.

Consistent with previous observations [30,48], the developed LFIA showed high concordance with the rSjSAP4-ELISA since both assays employed the same target antigen. Nevertheless, the results for a portion of samples remain inconsistent between the two tests. A total of 37 (6.74%) serum samples tested positive by the developed LFIA but negative by the rSjSAP4-ELISA, possibly because the LFIA probes all types of Abs against the target antigen, whereas the rSjSAP4-ELISA detects only IgG Ab. Conversely, 19 (3.46%) serum samples were positive according to the rSjSAP4-ELISA, but negative according to the LFIA test. Nevertheless, all these samples showed low OD and T/C values close to the cut-off threshold.

The incorporation of crude antigen lysate, such as soluble egg antigen (SEA) or somatic antigen, in the development of LFIA can cause cross-reaction to other parasitic flukes or soil-transmitted helminths [56,57,59]. For example, Rodpai *et al* [51] employed somatic extracts from adult *S*. *japonicum* as target antigens for the development of an immunochromatographic test (Sj-ICT), which showed cross-reactivity with flukes, such as *Opisthorchiasis viverrini*, *Clonorchiasis sinensis* and *Paragonimiasis heterotremus*. In contract, the employment of purified recombinant antigens in immunoassays helps lower the risk of cross-reactivity. Previously, it has been shown that rSjSAP4 has no cross-reaction with serum samples from patients diagnosed with alveolar echinococcosis and trichinellosis [60]. In addition, homology analysis revealed a low chance of SjSAP4 being cross-reactive with other parasitic flukes, such as *C*. *sinensis* and *Fasciola hepatica*, although this still needs to be validated with clinical samples [28]. Further, the present study preliminarily confirmed that the rSjSAP4-incorprated LFIA cassette has no cross-reaction with samples from individuals infected with hookworm, *A*. *lumbricoides*, and *T*. *trichuira*. Nevertheless, it is necessary to examine cross-reactivity with other helminths, particularly other trematodes, for the LFIA.

The present study has limitations: 1) Although the number of cases is low, participants with extremely high levels of rSjSAP4-specific Abs showed a less prominent control line on the LFIA cassette compared to those individuals with relatively low levels of anti-rSjSAP4 Abs. An optimization step, e.g., simultaneously dispensing a LM-labelled vertebrate-specific protein (e.g., his-tagged recombinant BSA) along with the LM-conjugated rSjSAP4 on the conjugate pad may help solve this issue. 2) Only samples collected from an area with moderate endemicity were tested with the developed immunochromatographic assay. Further evaluations of the assay as a monitoring/surveillance tool in LEAs of schistosomiasis japonica are needed. 3) Only serum samples were tested with the rSjSAP4-incorporatd LFIA cassettes. However, as a field applicable POCT, it should also work with plasma samples separated from a small volume of finger-prick blood, an approach which can make the whole test procedure more convenient and rapid.

## Conclusions

We developed an LM-based LFIA by incorporating rSjSAP4 and assessed its performance for the diagnosis of schistosomiasis japonica in a cohort residing in Barangay Ekiran, Leyte Province, the Philippines. With respect to the KK (+) individuals, the developed LFIA had a sensitivity of 80.6% and a specificity of 98.0%, which is comparable to the diagnostic performance of the previously developed GICA targeting the same antigen. The established LFIA and rSjSAP4-ELISA showed high consistency in the detection of *S*. *japonicum* infection. The prevalence of schistosomiasis japonica determined by these two methods was about 1.8 times greater than that obtained with the KK procedure performed on three stool samples. The developed LFIA can potentially be used as a powerful diagnostic tool for screening *S*. *japonicum* infection in humans and animal reservoirs in rural-endemic areas with limited access to equipment and supplies.

## Supporting information

S1 Table Descriptive data of the values used to build graph Fig 2. (XLSX)

S2 Table Descriptive data of the values used to build graph Fig 3. (XLSX)

S3 Table Descriptive data of the values used to build graph Fig 4. (XLSX)

S4 Table Descriptive data of the values used to build graph Fig 5. (XLSX)
